# Melatonin Ameliorates The Production of COX-2, iNOS, and The Formation of 8-OHdG in Non-Targeted Lung Tissue after Pelvic Irradiation 

**DOI:** 10.22074/cellj.2016.3857

**Published:** 2017-02-22

**Authors:** Reza Fardid, Ashkan Salajegheh, Mohammad Amin Mosleh-Shirazi, Sedigheh Sharifzadeh, Mohammad Ali Okhovat, Masoud Najafi, Abolhasan Rezaeyan, Akbar Abaszadeh

**Affiliations:** 1 Department of Radiology, School of Paramedical Sciences, Shiraz University of Medical Sciences, Shiraz, Iran; 2 Ionizing and Non-ionizing Radiation Protection Research Center, Department of Radiotherapy and Oncology, Shiraz University of Medical Sciences, Shiraz, Iran; 3 Diagnostic Laboratory Sciences and Technology Research Center, School of Paramedical Sciences, Shiraz University of Medical sciences, Shiraz, Iran; 4 Department of Biomedical Physics and Engineering, Faculty of Medicine, Tehran University of Medical Sciences, Tehran, Iran; 5 Department of Medical Physics, School of Medicine, Iran University of Medical Sciences, Tehran, Iran

**Keywords:** Radiation-Induced Bystander Effect, Melatonin, Cyclooxygenase-2, Inducible Nitric Oxide Synthase, 8-Hydroxydeoxyguanosine

## Abstract

In this study, we evaluated the bystander effect of radiation on the regulation of cyclooxygenase-2 (COX-2), inducible nitric oxide synthase (iNOS), and 8-hydroxydeoxyguanosine (8-OHdG) in lung tissues of Sprague-Dawley rats with and without pre-administration of melatonin. A 2×2 cm^2^ area of the pelvis of male Sprague-Dawley rats with and without pre-administration of melatonin (100 mg/kg) by oral and intraperitoneal injection was irradiated with a 3 Gy dose of 1.25 MeV γ-rays. Alterations in the levels of COX-2, iNOS, and 8-OHdG in the out-of-field lung areas of the animals were detected by enzyme immunoassay. The bystander effect significantly increased COX-2, iNOS, and 8-OHdG levels in non-targeted lung tissues (P<0.05). Melatonin ameliorated the bystander effect of radiation and significantly reduced the level of all examined biomarkers (P<0.05). The results indicated that the ameliorating effect of a pre-intraperitoneal (IP) injection of melatonin was noticeably greater compared to oral pre-administration. Our findings revealed that the bystander effect of radiation could induce oxidative DNA damage and increase the levels of imperative COX-2 and iNOS in non-targeted lung tissues. Interestingly, melatonin could modulate the indirect destructive effect of radiation and reduce DNA damage in non-targeted cells.

The radiation-induced bystander effect is the response in non-targeted cells or tissues by signals from nearby irradiated cells. Besides the direct effects of ionizing radiation in targeted cells, a series of biological effects can be induced in nonirradiated cells. These effects include apoptosis and cell death, chromosomal aberrations, DNA damage, mutagenesis, carcinogenesis, and an inflammatory response. For the first time, Nagasawa and Little (1) have reported this radiobiological phenomenon in November 1992. Since then, numerous related experiments have been conducted.

In radiotherapy, non-targeted effects may be related to the presence of secondary malignancies years after the treatment. It has been observed that a strong relationship exists between therapeutic radiation and secondary malignancies in patients. Secondary cancers may occur in patients who have under gone radiation therapy. Statistically, it has been estimated that there is a latency period of 5 to 15 years between exposure to therapeutic radiation and occurrence of secondary malignancies induced by radiation (2, 3). Several studies of patients treated by radiation therapy for prostate cancer have reported that lung cancer was one of the most critical secondary cancers which might be related to the bystander effect of radiation (4, 5). Local irradiation can increase the production of free radicals in other cells through secretion of cytokines such as transforming growth factor-beta (TGF-β), tumor necrosis factor-alpha (TNF-α), and interleukin-8 (IL-8) from the irradiated cells. These cytokines can induce different signals to the cells and alter the production and secretion of certain molecules via binding to the cell surface receptors. Therefore, they exert their influences on the regulation of the immune response, inflammation, and subsequent reactive oxygen species (ROS) and nitric oxide (NO) production (6). Calveley et al. (7) have observed that the level of inflammatory cytokines amplified in the apex of the lung due to irradiation of the lung base.

The genes involved in the formation of the bystander effect are often those involved in inflammation. Inflammatory cytokines such as TNF-α, TGF-β, IL-1, IL-6, IL-8, and IL- 33 induce cyclooxygenase-2 (COX-2) and inducible nitric oxide synthase (iNOS) gene expression, which lead to the production of NO and ROS (8).

COX-2 plays a significant role in the regulation of cell proliferation, survival, differentiation, and inflammation in addition to its role in initiation and progression of cancers (9). Recently, it was determined that COX-2 has physiological functions in the brain, kidneys, and cardiovascular system (10-12). Several studies have reported elevated levels of COX-2 in various types of cancers (10, 13). COX-2 is the leading cause of prostaglandin (PG) production such as PG-E2 and PG-I2, which contributes to free radical production (14, 15).

iNOS is another leading enzyme involved in the radiation-induced bystander effect and its level is under the control of a broad range of inflammatory cytokines (16). iNOS leads to a substantial amount of reactive nitrogen oxide species (RNOS) synthesis, which in turn contribute to some physiological and pathophysiological effects (17). Upregulation of NO synthesis is related to iNOS production, which is regulated through numerous pathways such as protein kinase C (PKC) and mitogenactivated protein kinase (MAPK) (18). Lymphocytes and macrophages have essential roles in the NO production, which increase the level of oxidative stress (19). It has been shown that after irradiation of a local area, derived NO from activated macrophage resulted in chromosomal damage, mutagenesis, suppression of mitosis, and apoptosis in nonirradiated cells (20, 21). *In vitro* examinations prove that free radicals are involved in the development of genomic in stability and bystander effect. NO is also proposed to be one of the main reasons for bystander effect due to its small size, hydrophobic nature, and ability to circulate freely within and between cells (22).

The study of DNA damage is essential and biomarkers of oxidative DNA damage are limited. In recent years, 8-hydroxydeoxyguanosine (8- OHdG) is recognized as a critical biomarker of oxidative stress and carcinogenesis. Oxidative guanine damage mostly occurs in the nuclear DNA (23). Results from animal trial studies have shown that oxidative stress leads to DNA damage and induction of 8-OHdG, which is crucial in mutagenesis and carcinogenesis (24).

Several studies report that lung tissue is susceptible to malignancies after radiotherapy (5, 25, 26). Lung tissue with plenty of macrophages is vulnerable to the radiationinduced bystander effect (27). Macrophages lead to iNOS production which contributes to COX- 2 induction. COX-2 is the leading cause of PG synthesis. PGs result in free radical production, and it has been revealed that increase of PGs can lead to lung cancer (28).

A common strategy for reduction of normal tissue toxicity against ionizing radiation is the use of immune modulators or radiation protectors. Administration of these agents before and after radiotherapy can modulate the response of normal tissues to radiation (29). In the past decade, the ability of melatonin in scavenging free radicals, especially hydroxyl radical (OH), is the primary reason to consider its radiation protection effects (30). Many studies have reported that melatonin (N-acetyl- 5-methoxytryptamin), the main secreted hormone of the pinealgland, is a potential direct and indirect free radical scavenger, an indirect antioxidant, and a strong immunomodulator (30-32). In this study, we evaluated the production of COX-2, iNOS, and formation of 8-OHdG in the out-of-field lung tissues of male Sprague- Dawley rats after 3Gy γ-ray administration to a designated section of the pelvic area of each animal. The modulatory effect of melatonin (oral and injection) on COX-2, iNOS, and 8-OHdG in the lung tissues was assessed after pelvic irradiation. In this interventional animal study, 49 male Sprague-Dawley rats that weighed 190- 210 g were purchased from the Laboratory Animal Resource Center of Shiraz University of Medical Sciences. The rats were kept in a room with a temperature of 22-24˚C and constant humidity, a12-hour light/12-hour dark cycle with adequate water and food. The room was disinfected by Sterl-STAT before starting the study. All tests were carried out 7 days after the new cage to create further adaptations of the animals to a new environment. This study followed the guidelines of the Declaration of Helsinki and the Ethics Committee of Shiraz University of Medical Sciences approved this study (Reference No. 93-8681).

The animals were randomly divided into 7 equal groups: control (without any treatment, drug administration, or irradiation), scattering radiation (whole-body irradiated with scattering dose), partial body irradiation (bystander group), oral administration of melatonin, intraperitoneal (IP) injection of melatonin, oral pre-administration of melatonin with partial body irradiation, and pre-IP injection of melatonin with partial body irradiation. According to previous studies (33, 34), we choose the dose of 100 mg/ kg of melatonin (Sigma, USA) to be administered to the rats in the related groups. Since the body weight was approximately 200 g, each animal received 20 mg of melatonin. Melatonin (20 mg) was dissolved in 1ml sterile double distilled water. Rats in the irradiation and sham irradiation groups received IP injections of melatonin 1 hour prior to irradiation. Likewise, 1 ml sterile double distilled water plus 20 mg melatonin was orally administered to the melatonin oral administration groups (irradiation and sham irradiation) 1 hour before irradiation. Each animal in the partial body irradiation groups was anesthetized by the injection of a combination of 0.2 ml ketamine (100 mg/ml, Sigma, USA) and 0.1 ml xylazine (20 mg/ml, Sigma, USA). A 2×2 cm^2^ area of the pelvis of each animal was selected and marked for irradiation. We used a Theratron Phoenix Cobalt-60 external beam radiotherapy unit (Best Theratronics, Canada) for irradiation. All rats in the partial body irradiation groups received a 3 Gy dose of 1.25 MeV 60Co γ-rays at a dose rate of 230 mGy per minute and source to surface distance (SSD) of 80 cm.

After 24 hours of irradiation, the rats were anesthetized, and their lungs were perfused and collected during surgery. All tissues were frozen immediately in liquid nitrogen and stored at -80˚C until processing.

In an attempt to differentiate the effect of scattering radiation from the bystander effect of radiation, we measured the amount of received scatter radiation using a Plexiglas rat phantom. First, dimensions of 10 rats were measured, and the mean was calculated by CorelDRAW software. A Plexiglas considered equivalent to the tissue was cut using a laser. Then a hole in the lung area of the phantom was created for the integration of an ionization chamber. Blocks of lead were located in the tray of the therapeutic head of the device to create the required 2×2 Cm^2^ field. A Plexiglas (about 5 cm thick) was placed in the back of the phantom as a backscatter layer. A 3Gy dose of 60Co γ-rays at a dose rate of 230 mGy per minute SSD of 80 cm was irradiated to the phantom by the Phoenix external beam radiotherapy unit (Best Theratronics, Canada). Regarding the room temperature and pressure, we measured the absorbed radiation in the lung area by a calibrated electrometer. The amount of measured radiation by the dosimeter was 7.5 mGy, which was applied to the whole body exposure of rats in the scattering radiation group.

We rinsed 100 mg of the lung tissue with 1X phosphate buffered saline (PBS), and homogenized the tissue with a T10 basic ULTRA-TURRAX Homogenizer system (IKA, Germany) in 1 ml of 1X PBS (pH=7-7.4). The tissue was stored overnight at -20˚C. After breaking the cell membranes by two freeze-thaw cycles, the homogenates were centrifuged for 5 minutes at 5000 x g and a temperature of 4˚C. The supernatant was removed and stored at -80˚C. All samples were centrifuged after thawing before the assay. Any repeated freeze-thaw cycles were avoided.

Levels of COX-2, iNOS, and 8-OHdG enzymes in the lung tissue homogenates were determined by Rat COX-2, iNOS, and 8-OHdG ELISA kits (Cusabio, China) according to the following instructions. First, each sample was diluted 1:6 by sample diluent solution and a 2-fold dilution series of the standard stock solution was produced. We added 100 μl of each standard and sample per well. The plate was covered with an adhesive strip and incubated for 2 hours at 37˚C. Next, the liquid of each well was removed and we added 100 μl of Biotin-antibody to each well. The plate was incubated for 1 hour at 37˚C. After incubation, the plate was washed three times with wash buffer. Then, 100 μl of HRP-avidin was added to each well and incubated for 1 hour at 37˚C. Next, the plate was washed five times with wash buffer. In the next step, 90 μl of Tetramethyl benzidine (TMB) substrate was added to each well and incubated for 15-30 minutes at 37˚C. Finally, 50 μl of Stop Solution was added to each well. We determined the optical density of each well within 5 minutes using a microplate reader set to 450 nm.

The gathered data were analyzed using SPSS V.20 software. The independent samples t test was used for comparison of the groups. All data were expressed as mean ± SD and P<0.05 were considered statistically significant.

According to the results, the iNOS level between the scattering radiation and the control groups was approximately equal at 191.41 ± 21.47 IU/ml (scattering radiation) and 189.66 ± 37.49 IU/ml (control). However, this measure for the bystander group was 13.6% higher than the control group (26.09 ± 16.14 IU/ml, P=0.132). We observed no statistically significant differences between the control, oral administration of melatonin, and IP injection of melatonin groups (P>0.05). We examined the iNOS levels in the groups which received pelvic irradiation. The iNOS level among the rats which were pre-administered oral melatonin had 22% less iNOS (P=0.041) and the pre-injected IP melatonin group had 29% less iNOS (P=0.007) compared to those who did not receivemelatonin. The IP injection of melatonin group showed significantly greater reduction in iNOS levels compared to oral administration of melatonin, with a mean difference of 13.8 ± 3.23 (P=0.04, Fig .1).

**Fig.1:**
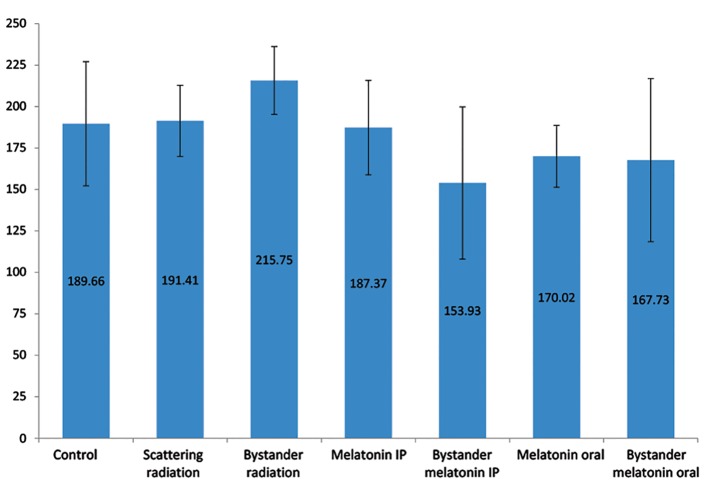
Comparison of inducible nitric oxide synthase (iNOS) levels in the lung tissue of rats from different experimental groups. Error bars indicate the SD of the mean for n=7 independent experiments. Oral administration and intraperitoneal (IP) injection of melatonin significantly decreased the iNOS levels by 22% (P=0.041) for oral administration and 29% (P=0.007) for IP injection. The reduction effect of the IP injection of melatonin was significantly more than the oral administration with a mean difference of 13.8 ± 3.23 (P=0.04).

Irradiation of the pelvic area significantly increased the COX-2 level in the lung tissue of the animals (54 ± 12.77 IU/ml, 25%) compared to the control group (P=0.004). Statistically, there was no significant difference between the control and the scattering radiation groups (P>0.05). We observed a noticeable drop (22%) in COX-2 levels of rats pre-injected with melatonin compared to the control group (P=0.013). Conversely, there was no statistically significant difference between the control and oral pre-administration of melatonin groups (Fig .2). In the groups that received partial body irradiation, pre-administration of melatonin meaning fully reduced the amount of COX-2 by 38% (P<0.0005) in the IP injection group and 20% (P=0.015) in the oral administration group. The effect of melatonin IP injection on COX-2 decline was significantly more than oral administration of melatonin, with a mean difference of 50.65 ± 13.55 (23% more effective, P=0.007, Fig .2).

**Fig.2:**
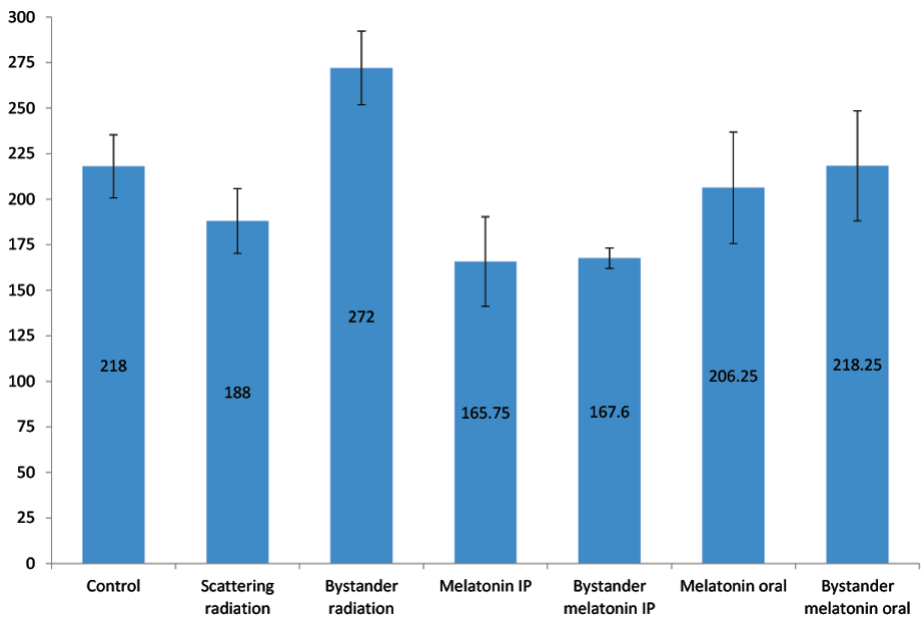
Comparison of cyclooxygenase-2 (COX-2) levels in the lung tissues of rats from different experimental groups. Error bars indicate the SD of the mean for n=7 independent experiments. Intraperitoneal (IP) injection and oral administration of the melatonin resulted in 38% (P<0.0005) reduction for the IP group and 20% (P=0.015) reduction for the oral group in COX-2 levels. The effect of the melatonin IP injection on decreased COX-2 levels was significantly more than oral administration, with a mean difference of 50.65 ± 13.55 (23% more effective, P=0.007).

The results revealed that bystander radiation caused DNA damage and significant elevation of 8-OHdG, an increase of 13.74 ± 4.71 (27%) compared to the control group (P=0.013). The 8-OHdG level in the scattering radiation group (52.77 ± 8 IU/ml) was roughly equivalent to the control group (51.39 ± 11.61 IU/ml).

Melatonin administration (IP injection or oral) had no significant effect on the 8-OHdG levels compared with the control group (P>0.05). Melatonin neutralized the impact of the radiation bystander effect. The level of 8-OHdG decreased by 37% (IP injection) and 34% (oral) in these rats (P<0.0005). The IP injection of melatonin more efficiently reduced the 8-OHdG level compared to the oral administration of melatonion (P=0.045, Fig .3).

Our findings have shown that administration of melatonin could modulate increased COX-2 and iNOS synthesis and reduce DNA damage related to the radiation-induced bystander effect. These enzymes have an important role in inflammatory pathways and oxidative damage after exposure to radiation. The presence of harmful bystander signaling from cancer radiation therapy may be involved in the long-term outcomes of radiation therapy such as the incidence of secondary lung cancer (15). Studies have shown that both COX- 2 and iNOS have a fundamental role in oxidative damage and mutation in non-irradiated cells. Over expressions of both COX-2 and iNOS are associated with a higher tumor growth rate and lower survival rate (28, 35, 36).

**Fig.3:**
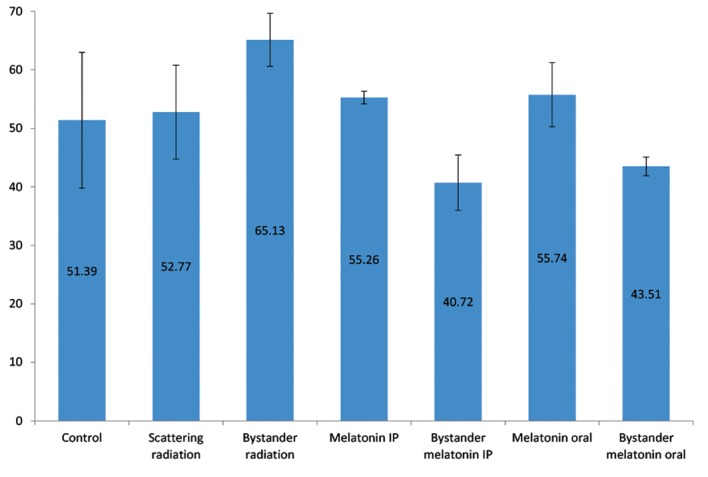
Comparison of 8-hydroxydeoxyguanosine (8-OHdG) levels in the lung tissues of rats from different experimental groups. Error bars indicate the SD of the mean for n=7 independent experiments. Melatonin significantly decreased the level of 8-OHdG by 37% in the intraperitoneal (IP) group and 34% in the oral administration group (P<0.0005). The IP injection of melatonin more efficiently reduced 8-OHdG levels compared to the oral administration group (P=0.045).

In this study, pelvic irradiation resulted in upregulation of COX-2 by 25% in non-targeted lung tissue, which in turn contributed to oxidative DNA damage and induction of 8-OHdG (by 27%). Chai et al. (37) also observed that lower abdominal irradiation induced COX-2 expression in the bronchial epithelial cells of non-targeted lung tissues, which ledto the concurrent increase in prostaglandin production and oxidative DNA damage. In their study, the peak time for induction of COX-2 was 24 hours after partial abdominal irradiation. Direct irradiation can cause upregulation of COX-2 and PG-E2 synthesis in different radiation doses, which results in decreasing viability. However, it may not affect the cell cycle and apoptosis (38). Secretion of cytokines from irradiated cells can induce COX-2 and iNOS synthesis in non-irradiated cells.

According to the results of our study, irradiation of the pelvic area significantly increased the iNOS level in the lung tissue of the animals by 13.6%. However, pre-injection of melatonin counteracted the bystander effects and the iNOS degree in the non-targeted tissue reduced by 29%. Ghosh et al. (39) described upregulation of iNOS and other inflammatory genes (e.g., NFKB1) in the cells due to the bystander effect of partial irradiation. iNOS elevation was associated with increased NO production, DNA damage, and apoptosis. Treatment with L-NAME (NOS inhibitor) reduced DNA damage related to the bystander effect.

There is a relationship between upregulation of COX-2 and high-risk malignancies (40). Prostaglandins can trigger ROS synthesis, oxidative DNA damage, and mutagenesis such as the formation of 8-OHdG (41). In addition to DNA damage induced by NO, upregulation of NO production can inhibit BRCA1 expression with a subsequent downregulation of DNA repair pathways and shift to the error-prone NHEJ. These result in malfunction of the DNA repair process that leads to genomic instability and a higher risk of cancer (42).

Strategies for management of COX-2 and iNOS production may represent a useful biological response for both normal and tumor tissues in patients that receive radiotherapy. Chai et al. (43) have reported that inhibition of the TGF-β/COX-2 pathway can cause resistance to DNA damage in out-of-field lung tissue. Previous studies have proven that melatonin has a protective effect via inhibition of NFKB1 activation and reduction of COX-2 and iNOS expression (44, 45). Likewise, this may be mediated by suppression of p53 acetylation (46). Our results have indicated that pre-administration of melatonin neutralized the bystander effect of irradiation in the out-of-field tissue and significantly decreased levels of iNOS by 29%, Cox-2 by 38%, and 8-OHdG by 37%.

Karbownik et al. (47) showed that melatonin completely counteracted the effects of ionizing radiation. They observed that an IP injection of melatonin (50 mg/kg) protected guanine bases in DNA from oxidation and reduced the 8-OHdG level in the liver tissue of rats that received whole body ionizing radiation. Similarly, our findings showed that an IP injection of melatonin reduced the 8-OHdG level by 37% in the out-of-field lung tissue and neutralized the DNA damage related to the radiation bystander effect.

COX-2 and iNOS are two important factors in oxidative damage induced by ionizing radiation. Both are overexpressed during chronic inflammation and may be involved in subsequent cancer occurrences long-term after exposure to radiation.

In this study, we investigated the amelioration of COX-2 and iNOS overexpression in the out-of-field lung tissue by pre-administration of melatonin. We irradiated a local 2×2 cm^2^ area of the pelvis, which simulated radiotherapy conditions. We determined the protein levels of COX-2 and iNOS in the out-of-field lung tissue 24 hours after partial pelvic irradiation with and without pre-administration of melatonin. Karbownik et al. (47) showed that the protein level of COX-2 reached a peak 24 hours later in the non-targeted lung tissue. Consequently, COX-2 induction resulted in increased prostaglandin and ROS production that led to oxidative DNA damage. Our ELISA analysis showed a significant increase in the levels of COX-2, iNOS, and 8-OHdG in non-targeted lung tissue. We compared the scattering radiation group with the bystander group and confirmed that scattering radiation did not have any significant effects on COX-2 and iNOS levels, and formation of 8-OHdG.

Our findings proved that pre-administration of melatonin could modulate COX-2 and iNOS levels and counteract any potential oxidative DNA damage in out-of-field lung tissue after partial body irradiation. We observed that the IP injection of melatonin was significantly more efficient in ameliorating the bystander effect compared to oral administration of melatonin.

A better understanding of the mechanisms involved in the radiation-induced bystander effect would be appreciated to consider essential measures that manipulate probable oxidative damage in normal tissue and increase the therapeutic gain in radiotherapy.
